# Do free healthcare policies play a role in expanding national health insurance enrollment among informal sector workers? The case of the Afya Care pilot program in Kenya

**DOI:** 10.1186/s12889-025-24760-3

**Published:** 2025-10-27

**Authors:** Phidelis N. Wamalwa, Christoph Strupat, Edmund Yeboah, MaryBennah N. Kuloba, Boniface Mbuthia, Manuela De Allegri

**Affiliations:** 1 Medical Faculty, Heidelberg Institute of Global Health, Heidelberg University, Im Neuenheimer Feld 365, Heidelberg, 69120 Germany; 2 German Institute of Development and Sustainability (IDOS), Bonn, Germany; 3 African Medical and Research Foundation, Kenya Head Office, Nairobi, Kenya

**Keywords:** Universal health care, Free healthcare policies, National Health Programs, Health insurance, Informal sector

## Abstract

**Background:**

Several Sub-Saharan African countries have transitioned from fiscally unsustainable free healthcare policies to national health insurance schemes. These national schemes often expect informal sector workers to contribute directly to the scheme. Although enrollment in the national scheme is critical to achieving universal health coverage, informal sector workers’ inclusion remains challenging. In this paper, using the case of the Afya-Care free healthcare policy pilot in Kenya, we examine an issue that has thus far received limited attention in the literature: the extent to which earlier exposure to free healthcare policies shapes later informal sector workers’ decisions to enrol and contribute to a public health insurance scheme.

**Methods:**

We used nationally representative repeated cross-sectional household data collected among informal sector workers in Kenya in 2018 and 2020 before and after the Afya Care-free healthcare pilot, respectively. Using a sample of 6900 informal sector workers, we constructed a pre-and-post quasi-experimental design applying difference-in-differences estimation modelling to assess the effect of exposure to the Afya-Care free healthcare policy pilot on individuals' decisions to enrol later in the national health insurance scheme.

**Results:**

No Difference was observed between the two groups before intervention. However, after the intervention, our findings show a 10.5% difference in national health insurance scheme enrollment between the control and intervention groups (*p* < 0.001). Individuals in the intervention counties had a 65% greater probability of enrolling in the national health insurance scheme than did those in the control counties (odds ratio -1.65; *p* = 0.07; 95% CI—0.96- 2.83). Heterogeneity analysis revealed a significant increase in enrollment in the intervention group by 30% and 18% among the least educated and among the poorest individuals, respectively.

**Conclusions:**

Our findings suggest that exposure to the Afya Care-free healthcare policy favoured the decision to enrol in the national health insurance scheme and enhanced equity. However, registration in the scheme remains low. Increased enrollment is likely to follow only from an enhanced government commitment to improve information campaigns to boost awareness, to expand subsidies to increase premium affordability, especially among low-income informal sector workers, and to subsidise those who cannot afford to pay.

**Supplementary Information:**

The online version contains supplementary material available at 10.1186/s12889-025-24760-3.

## Background

In the wake of the Universal Health Coverage (UHC) vision, a well-functioning health financing system should ensure that a population has access to quality healthcare services when needed without facing financial hardships [1, 2]. Instead, the 2023 UHC progress report highlights how most Sub-Saharan African (SSA) countries may fall short of meeting the sustainable development goal 3.8, which aims to achieve UHC, including financial risk protection, access to quality essential health services, and access to safe, effective, quality, and affordable essential medicines and vaccines for all, by 2030 [1, 3]. By its definition, UHC aspires to leave no one behind, but its implementation, especially in SSA, faces challenges ranging from fragile health systems to inadequate financing, governance, and leadership [4]. Achieving UHC requires countries to invest in strengthening their health systems to provide a full continuum of affordable essential health services, especially to disadvantaged populations.

Many SSA countries have experimented with different health financing strategies, such as free health care (FHC) and social health insurance policies, to accelerate progress toward achieving Sustainable Development Goal (SDG) 3.8 [5–10]. Although different in their application, almost all recent FHC reforms aim to remove or at least drastically reduce payments at the point of use, shifting toward prepaid, pooled, and/or subsidised payment mechanisms [6]. Over the last decade, publicly funded health insurance schemes have expanded rapidly across SSA [11]. These schemes normally build on the original contributory infrastructure of a scheme already in place for people employed in the formal sector, expanding coverage to more population groups by directing government subsidies into the scheme [12]. The challenge arises from the fact that these direct government subsidies are normally reserved for selected population groups, for instance, pregnant women, children, or individuals living in poverty [13, 14]. As a result, informal sector workers (ISWs) are often expected to pay their contribution.

By definition, the informal sector encompasses all economic activities by workers and economic units that are in law or practice not covered or insufficiently covered by formal institutional arrangements, excluding illicit or economic activities that are forbidden by law [15]. The absence of formal or poorly developed employer-based structures in the informal sector limits the administrative capacity to apply the needed check-off systems, mechanisms through which employers deduct premiums directly from salaries and transfer them to the scheme on behalf of the ISWs [16, 17]. This imposes a barrier to health insurance coverage expansion among ISWs. Furthermore, ISWs are characterised by insecure nonpayroll employment, making it difficult to assess income for a premium deduction, and they earn low and unpredictable incomes that limit premium affordability [2, 18].

Additionally, ISWs are usually left to freely decide whether to join or not join a scheme. However, the literature has clearly shown that voluntary enrollment advances adverse selection and inequity, undermining the very purpose of UHC [19, 20] Moreover, ISWs, by the nature of their work and living conditions, are at a greater risk of illness and have a lower probability of being able to afford health care [21, 22]. Therefore, ISWs remain a critical population of focus to achieve UHC due to the health vulnerabilities they face and the massive size of the employment sector in many SSA countries.

Evidence from the literature shows numerous benefits of health insurance, including but not limited to a reduction in catastrophic expenditures and out-of-pocket payments [23]. In Kenya, health insurance uptake and demand remain relatively low, with only approximately 19% and 26% of the population covered as of 2018 and 2022, respectively [24, 25]. The main insurer, the National Health Insurance Scheme (NHIS),^1^ was founded in the 1960 s, initially with a limited scope, covering only formal sector workers, until 1998, when the scope expanded to offer both inpatient and outpatient coverage to all formal sector workers, and in 2015, the inclusion of ISWs [12, 26–28]. Until 2020, the scheme was funded through compulsory contributions from formal sector workers, voluntary contributions from ISWs and the unemployed, and government subsidies for certain vulnerable groups and services [14, 16, 26, 28]. Despite these efforts, health insurance coverage is not only low but also inequitably distributed, with only 8% of ISWs, who constitute 84% of the employment sector, enrolled. This trend leaves most of the ISWs with no access to affordable healthcare services [26, 29].

To increase access, the Kenyan government commissioned a one-year free healthcare policy pilot, the "Afya Care" UHC pilot (hereafter referred to as the AC-FHC pilot) project. It was implemented between December 2018 and June 2020 in four of the 47 counties, removing user fees and hence making health services free for all in the four counties. The government aimed to learn from the process and scale up the project to the rest of the country [30, 31]. Based on the lessons learned from the pilot’s implementation and performance review, and considering the volatile nature of government revenue and the insufficient allocation of resources to the healthcare sector, maintaining a nationwide free healthcare system sustained via taxation appeared unfeasible [32]. As a result, the government opted to encourage mandatory enrollment for all into NHIS as the main vehicle to achieve UHC by 2030 [30, 33]. However, transitioning a population from free or fully subsidised services to mandatory insurance schemes can be challenging, especially for individuals not in formal employment. If a country lacks the necessary institutional structures to enforce compulsory contributions, it often results in many economically disadvantaged groups, such as ISWs and the unemployed, and those from vulnerable households being left uncovered and with difficulties accessing healthcare services [34–36].

Kenya, however, is not the only SSA country that has made a transition from an FHC policy to a national insurance scheme. Substantial evidence from the literature shows that several SSA countries have transitioned from publicly funded healthcare [9, 37–39] and, more recently, from FHC policies [10, 40–42]. For instance, in Zambia, before transitioning to the NHIS in 2018, all health services in primary health services were offered for free [10, 43]. Similarly, in Burkina Faso, the nationwide gratuité policy, which abolished user fees for pregnant and lactating women and children under five, preceded the establishment of the universal health scheme in 2023 [41, 42]. For Ghana, the introduction of selected maternal care user-fee-removal policies, which were later integrated into the NHIS, preceded the publicly subsidised insurance scheme [37, 38, 40]. Despite the increasing transitions from free or subsidized health policies to NHIS, where individuals or households are expected to enroll and pay premiums to access the same services they previously received for free, there is limited evidence of how prior exposure to free or subsidized policies, implemented outside the NHIS or with no prior intention to shift to NHIS shapes individuals’ decisions to later enroll in the NHIS among the ISWs. The evidence available in the literature from several experimental studies across low and middle-income countries focuses either on the effect of subsidies on service utilisation and out-of-pocket expenditures [44–47] or on subsidising health insurance premiums (within NHIS structures) [34, 44]. For instance, a study in Ghana reported an increase in facility deliveries and found that insurance coverage among pregnant women increased substantially by 17.5% when insurance premiums were fully subsidised compared with 1.4% before subsidisation [44]. Several experimental studies in SSA and Asia reported mixed findings regarding implementing temporary periods of free premium subsidies, time-limited registration drives, and targeted educational programs as a way of increasing both enrollment and retention in NHIS [23, 34, 45–48]. For all these studies, however, the subsidies were within the NHIS structures.

From a theoretical perspective, exposure to free services provides an opportunity to experience a service that one would otherwise not experience if the cost was attached. Individuals' experiences with free or subsidised health policies, whether positive or negative, influence their decision to pay or not pay for a service or its best replacement; in this case, NHIS premiums once free/subsidised services are stopped. This approach has been widely and successfully applied in preventive health products, such as water, sanitation, and hygiene, to create demand for uptake [49–51]. Nigel and Acharya, in their scoping review, constructed a demand curve for health insurance programs based on information extracted from four countries (Vietnam, Burkina Faso, Ghana, and Indonesia) [34, 45, 46, 48]. They found a consistent trend, as reported in other health products, with health insurance uptake increasing when individuals were offered a higher premium subsidy [48]. However, whether exposure to nonpremium subsidies motivates individuals to enrol later in insurance schemes remains unknown. Our study contributes to filling the existing knowledge gap by focusing on ISWs. We aimed to evaluate how exposure to the AC-FHC pilot shaped informal sector workers’ decisions to later enrol in the NHIS in Kenya.

## Methodology

### Study setting

Kenya is a lower-middle-income country with an estimated population of 47 million, with approximately two-thirds living in rural communities [52] and a large informal employment sector accounting for 89% of all employees [24] The Kenya health system is organized around four hierarchy tiers that are based on the type of health services delivered: community (Level 1); primary care facilities (Levels 2–3); sub-county (primary) and county referral (secondary) hospitals (Levels 4–5); and national teaching and referral (tertiary) hospitals (level 6) [53]. Following devolution in 2010, the Kenyan health system was organised into two main functional levels: the national level through the Ministry of Health, focusing on policy formulation and regulation, and the regional level through 47 semi-autonomous county governments responsible for coordinating and managing the delivery of primary and secondary healthcare services, including health financing [54].

Health is mainly tax-funded (46%), complemented by donor funding (19%) and private funding (35%), which includes health insurance premiums and out-of-pocket payments [53]. The NHIS is the main health insurance scheme in Kenya, accounting for approximately 92% of total coverage [25]. Enrollment is largely driven by the formal sector, which makes up 80% of NHIS enrollees, while ISWs, constituting 89% of the employed population, make up only 8% of NHIS coverage [24–26]. For formal sector workers, contributions are deducted directly from their salaries through a graduated scale based on income levels, ranging from Kenyan shillings (KES) 150 to 1,700 (USD 1.15 to 13.08) per month [16]. In contrast, since the inclusion of ISWs in the NHIS as voluntary contributors, the premium has been maintained at a flat rate contribution of KES 500 (USD 3.85) per month [7, 14, 16, 27]. The flat rate is often considered unaffordable by many ISWs and inequitable because it does not account for varying income levels in the informal sector [7, 16]. Although NHIS enrollment for ISWs became mandatory under the amended 2022 NHIS Act [30, 33], the government has yet to implement specific strategies to address the barriers and encourage enrollment.

### Description of the intervention

**Fig. 1:**
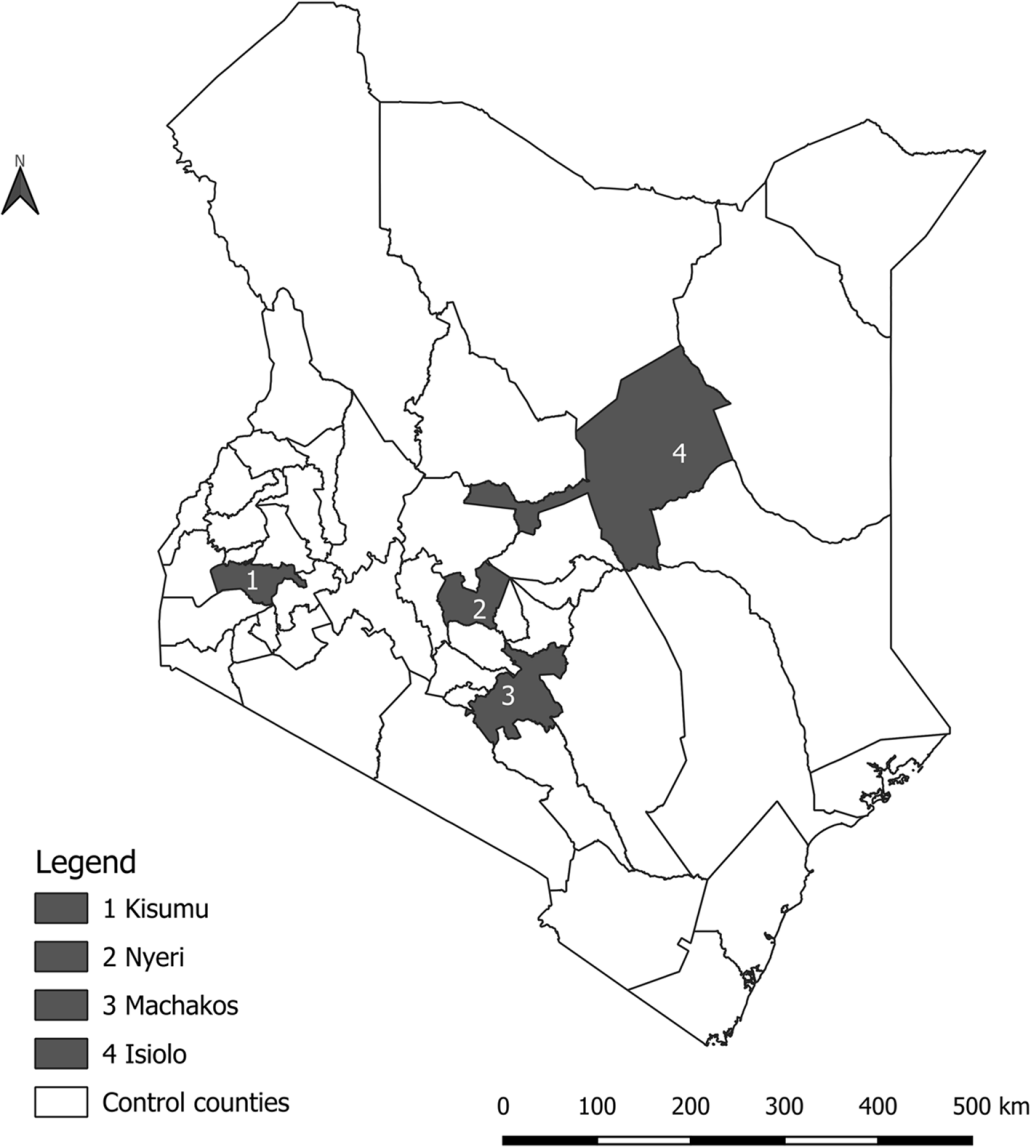
Map of Kenya showing the intervention counties

In December 2018, the national government launched and implemented the AC-FHC policy in four purposively selected counties: Kisumu, Nyeri, Isiolo, and Machakos, intended to improve the utilisation of needed health services by reducing financial barriers to access [30, 31]. Figure 1 highlights the four intervention counties on the map of Kenya. The pilot was planned to be implemented for 12 months (December 2018 – December 2019). However, there were some variations in the actual implementation time between counties, with implementation in some counties running until June 2020.

The counties were selected to represent diverse disease burdens in the country and other important demographic characteristics, aimed at achieving a geographical and political balance [55, 56]. Table 1 below highlights selected characteristics of the pilot counties.

**Table 1:** Health system, socio-economic, and demographic characteristics of the pilot counties

Characteristic [reference]	Kisumu	Nyeri	Machakos	Isiolo
Population [57]	1,155,574	759,164	1,421,932	268,002
Poverty headcount rate (%) [58]	39.0	26.0	38.3	55.6
Health system profile [59–62]	210 health facilities (HFs)	366 HFs:	367 HFs:	47 HFs:
	• Public: 125	• Public: 118	• Public: 198	• Public: 30
	• FBO: 15	• FBO: 19	• FBO: 32	• FBO: 9
	• NGO: 17	• NGO: -	• NGO: 9	• NGO: 1
	• Private: 53	• Private: 229	• Private: 128	• Private: 7
Health insurance coverage -% [24]	18.1	41.8	18.9	11.1
Selection criteria [30, 56]	Densely populated, high HIV and malaria burden	High levels of NCDs	High rates of traffic accidents	Arid county, sparse normadic population, and high mortality rates

We found no literature on the implementation fidelity of the AC-FHC pilot; anecdotal evidence, however, suggests that since counties are semi-autonomous entities, actual implementation processes may have differed slightly across counties. The AC-FHC pilot was characterised by the removal of user fees at levels four and five hospitals, strengthening health systems (supplies, human resources, and equipment), supply of medicine through the Kenya Medical Supplies Agency (KEMSA), and strengthening community health systems. With health services already fully subsidised in level two and three health facilities, all individuals in the AC-FHC pilot counties had an opportunity to access health services for free in all public health facilities based on the UHC benefit package. However, to prevent spillovers to neighbouring counties, each household was expected to be registered and issued an AC-FHC card to access services.

The registration was conducted at no cost at the household level by community health volunteers (CHVs), at a nearby health facility, or in various community mobilisation areas temporarily set up for registration. A total of 3.1 billion Kenyan shillings (~ USD 23.9 million) was invested in financing the AC-FHC pilot, with 88% allocated to the governance and improvement of service delivery on the supply side; 70% of this allocation was for KEMSA. The remaining 12% was allocated to community health services for training and equipping CHVs on the demand creation side [31, 63].

### Study design

We adopted a pre-post quasi-experimental design with independent controls to assess the effect of the AC-FHC pilot on the later probability of an individual's decision to enrol in the NHIS among ISWs. For our analysis, we identified the four AC-FHC pilot implementation counties where all services were free as the intervention group and 42 out of the remaining 43 counties as the control group.^2^ To assess the effect, we compared health insurance enrollment (outcome) among ISWs residing in the intervention counties (exposed) and control counties (nonexposed), making use of data collected at two different time points before and after the AC-FHC pilot launch.

### Data sources

We used secondary data from two nationally representative in-person repeated cross-section surveys among informal sector households conducted in Kenya by the Friedrich-Ebert-Stiftung, the International Labour Office, and the Institute for Development Studies at the University of Nairobi in collaboration with the German Institute of Development and Sustainability (IDOS). The first data collection round took place in November/December 2018, just before the launch of the AC-FHC pilot, and the second data collection round took place in December 2020, a few months after the AC-FHC pilot ended. For this analysis, the authors received the data in an aggregated and fully anonymised format, now available in a public repository [64].

For sensitivity analysis and to meet the difference-in-differences (DID) analysis assumptions, we used common trend analysis, which requires more than two pre-intervention data points. Our main study data had only one data point, so we obtained additional data from the 2013 and 2018 Kenya Household Health Expenditure and Utilisation Surveys. This dataset was the only available nationwide household data with health insurance coverage estimates by county and employment status at the time of the study.

### Data collection procedures

The sample frame associated with our survey included all households in Kenya that operated in the informal economy on the day of the survey. To ensure every household that operated in the informal economy had an equal opportunity for inclusion in the sample, a clustered, stratified, multi-stage, probability sampling design was used. This guaranteed that the survey yielded a representative estimate of the perspectives of the target population. To achieve this, random selection methods were strictly applied at every stage of sampling, with the probability of sampling proportionate to the adult population size.

To identify households operating in the informal economy, in-person interviews were conducted in two phases at each household. The first phase of the interview involved the household head providing demographic and employment information on each member (15 or older) within the household. A list of household members operating in a formal and informal economy was created. If at least one member was active in the formal economy, the household was replaced. If no member was active in the formal economy, the respondent was randomly selected from the list of people operating in the informal economy for the second part of the interview. This study utilises data from in-person interviews conducted with the household head, who provided information, including the health insurance enrollment status of all household members.

The sample size was determined by proportional random sampling based on the share of the national population. The selected sample was determined by random selection methods at every sampling stage, and the application of probability sampling was based on population data. A publication by Strupat (2022) provides a detailed description of the sampling design and sampling process [65]. In total, 1,188 households (in 2018) and 2,608 households (in 2020) were sampled for interviews. Considering the applicable legal age to join the NHIS voluntarily, only individuals 18 years and older were included in the analysis.

### Conceptual and theoretical framework

We set our work against a conceptual model theoretically inspired by the free trial approach. This customer acquisition model leverages the concept of offering individuals a cost-free service for a limited period to enable them to experience the benefits of that service without financial risk and value it before being asked to purchase it [50, 51]. In the health sector, this principle has mostly been applied to health prevention products. A study by *Tsai *et al*.* on the effect of exposure to water treatment products on the repurchase rate [50] and that of *Dupas* evaluating the effect of short-run subsidies on Olyset-treated insecticidal nets on the long-term willingness to pay [49] provide concrete evidence in support of the free trial approach in the public health field. In both studies, short-term exposure to the subsidised service and products positively impacted the willingness to pay for the treated net or water treatment product after the subsidy ended, to continue experiencing the benefits [49, 50]. In the health insurance industry, this principle is increasingly being applied in policies that offer temporary health insurance premium subsidies to promote enrollment [23, 34, 45–48]. The initial period of reduced or no-cost enrollment serves as an entry point, allowing individuals to experience coverage benefits without immediate financial commitment. Positive experiences during the subsidised period may motivate individuals’ willingness to pay for insurance once the subsidy ends.

In our study, the AC-FHC policy allowed ISWs to access and experience free healthcare services. We assess how the experience may have influenced their decision to later enrol in the NHIS. We postulate that ISWs who experienced the benefit of accessing services at no cost at the point of care, thanks to the AC-FHC policy, may be more likely to join and pay NHIS premiums. We assume they may view it as an enabler to continue experiencing health services without incurring substantial financial losses due to high out-of-pocket expenses. Consequently, we hypothesise that after the AC-FHC pilot ended, the intervention counties may experience higher NHIS enrollment rates among ISWs than the control counties, owing to the influence of the experience with the AC-FHC policy.

### Definition of variables

Table 2 below summarises the variables included in the study. The outcome variable, health insurance enrollment, is dichotomous and defines whether an individual is enrolled in the NHIS (1) or not (0). The exposure variable is defined as a dummy variable, indicating whether an individual resides in the intervention (1) or control (0) county. To improve the precision of the estimates, we included the following individual and household-level variables in our estimation model: location (rural/urban), age, sex, household size, employment sector, the wealth asset index, and the education level of the household head. The wealth quantiles were calculated from the list of assets reported by a household using principal component analysis as a cumulative weighted index. The index was further divided into four groups to capture wealth distributions across quartiles (“Lowest quartile”, “Lower-middle”, “Upper middle”, and “Highest quartile”).

**Table 2:** Definition of variables

Variable	Definition	Measurement
**Outcome**		
Health insurance enrollment	Individuals who reported being enrolled in the national health insurance scheme	0 = No
		1 = Yes
**Exposure**		
‘Afya Care’ free health care policy	Being a resident in an intervention or control county	0 = Control
		1 = Intervention
**Covariates**		
Age	The age of the individual in years	18–34 years
		35–54 years
		> 54 years
Sex	Sex of individuals	Male
		Female
Household size	The total number of members in a household	Small < 3
		Average 4–6
		Large > 6
Location	Household location	Urban
		Rural
Education	The educational level of the household head	No basic education
		Completed primary education
		Post-primary education
Employment sector	The informal employment sector, where individuals work	Unemployed
		Agricultural sector
		Non-agricultural sector
Wealth asset quantiles	It was calculated from the list of assets reported by a household as a cumulative weighted index	1 = Lowest quartile
		2 = Lower-middle
		3 = Upper middle
		4 = Highest quartile

### Data analysis and empirical approach

We used descriptive statistics (chi^2^ and T-test) to test differences in sample distribution characteristics between the intervention and control counties, including enrollment rates between intervention and control counties before and after the intervention. In addition, a bivariate descriptive heterogeneity analysis was conducted among ISWs enrolled in NHIS in the intervention and control groups, before and after the policy implementation. To assess the effect of the AC-FHC pilot on health insurance enrollment, we constructed a difference-in-differences (DID) logistic regression model. The intervention and time interaction output indicates the average treatment effect of the AC-FHC policy. The model was based on the following specifications [66]:

1 $$\begin{aligned} {\mathrm Y}_{\mathrm{ict}}\;=&\;{\upbeta}_0\;+\;{\upbeta}_1\mathrm{AC}-{\mathrm{FHC}}_{\mathrm{ct}}\;+\;{\upbeta}_2{\mathrm{Time}}_{\mathrm t}\;\\&+\;{\upbeta}_3(\mathrm{AC}-{\mathrm{FHC}}_{\mathrm{ct}}\;\ast{\mathrm{Time}}_{\mathrm t})\;\\&+\;{\upbeta}_4{\mathrm X}_{\mathrm{it}}\;+\;{\mathrm V}_{\mathrm c}\;+\;{\upvarepsilon}_{\mathrm{ict}}\; \end{aligned}$$

Where *Y*_*ict*_ is the outcome of interest for whether individual (*i*) living in the county (*c*) had insurance at a given time (*t)*. *ß*_*0*_ is the constant term. *AC-FHC*_*ct*_ is the intervention dummy variable indicating whether the county (*c)* was exposed to free healthcare services (= 1) or not (= 0) and at a given time (*t*). Time is a dummy variable indicating before (= 0) and after (= 1) the intervention. The difference-in-differences estimator is then given by the interaction of the time and intervention dummy, hence '*AC-FHC*_ct_*Time_*t*_*',* with its corresponding coefficient *ß*_*3*_. X_*it*_ represents a set of individual and household characteristics observed during each survey. To account for the Different initial development levels of the counties that are possibly related to the outcome variable and the AC-FHC pilot policy, we include county fixed effects for the 46 county dummies shown by *V*_*c*_. Since we dealt with repeated cross-sectional survey data and a larger sample in the second wave, we clustered the standard errors at the survey wave-county level by interacting the 47 county dummy variables (V*c*) with the survey round indicator *Time*. The time-variant heterogeneity between the counties ensures that county-specific changes between the first and second rounds of the survey do not directly drive the findings.

To ensure the validity and reliability of the DID approach, two crucial assumptions are essential: the no spillover effect and the parallel trends assumption (PTA). Before commencing the DID model analysis, we tested these assumptions for our data. The spillover effect assumption that ensures the treatment of the intervention group does not affect the control group was effectively controlled at the intervention design stage. Residents of the pilot counties had to register and be issued a special access card, which was not accessible to the neighbouring counties. The parallel trends assumption requires that in the absence of treatment, the average outcomes for the intervention and control groups would have followed parallel paths over time [66]. We conducted this assessment using a visual inspection of pre-intervention trends by plotting the average outcome variable over time for both groups to examine whether the trends were parallel before the intervention. We then formally estimated a pre-trend regression model statistical test, including interaction terms between time dummies and the treatment group for periods prior to the intervention. PTA holds if the lines are roughly parallel with visual inspection or the absence of statistically significant coefficients on these interaction terms, supporting the validity of the DID model.

**Fig. 2:**
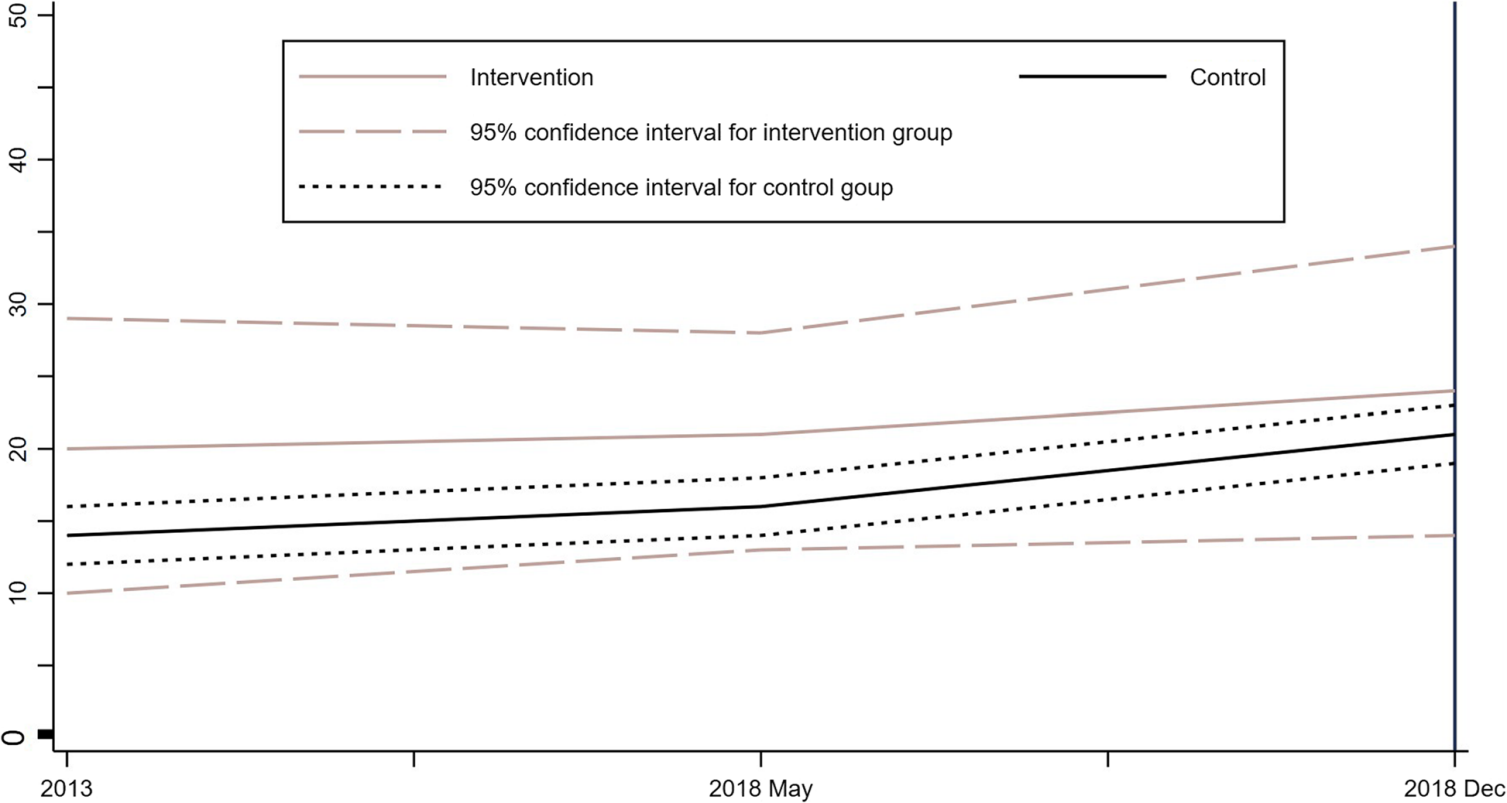
Parallel trend analysis

Figure 2 presents the parallel trends in health insurance coverage in the intervention and control groups from 2013 to December 2018, before the implementation of the AC-FHC policy. The graph indicates that health insurance enrollment followed parallel paths before the intervention, confirming the parallel trends assumption. Pre-trend regression results show no differential trends before the intervention (*p* = 0.187), confirming that the parallel trends assumption holds. The parallel nature of these trends suggests that the DID estimates could reflect the causal impact of the AC-FHC policy on health insurance enrollment. However, we focus on emphasising associations rather than asserting causality due to the reliance on repeated cross-sectional data. The intervention counties display a large 95% confidence interval due to their small sample size.

## Results

Our analysis included 6900 individuals (2354 in 2018 and 4546 in 2020). Table 3 shows the sample characteristics and NHIS enrollment rates among ISWs in the intervention and control groups before and after the AC-FHC pilot implementation. Overall, approximately half of the participants were between the ages of 18 and 34 years and came from households of average size (3–5 members). In both groups, two-fifths of the respondents were working in non-agricultural sectors. Approximately two-thirds of the respondents in the control counties were based in rural areas, while in intervention counties, about half were living in rural areas.

**Table 3:** Sample descriptive statistics by exposure group and year

Variables	Survey 1-baseline (Before intervention)			Survey 2 (After intervention)		
**Exposure group**	**Control**	**Intervention**		**Control**	**Intervention**	
**Number of observations**	**2131**	**223**		**4235**	**311**	
	**%(n)**	**%(n)**	**Difference**	**%(n)**	**%(n)**	**Difference**
Insurance coverage						
Yes	11.7 (250)	15.2 (34)	−3.5	11.7 (496)	22.2 (69)	−10.5***
No	88.3 (1881)	84.8 (189)		88.3 (3739)	77.8 (242)	
Age						
18–34 years	57.5 (1226)	52.9 (118)	4.6	54.6 (2315)	49.2 (153)	5.4*
35–60 years	36.9 (786)	39.5 (88)	−2.6	37.7	39.5 (123)	−1.8
> 60 years	5.6 (119)	7.6 (17)	−2.0	(1596) 7.7 (324)	11.3 (35)	−3.6**
Sex						
Male	52.1 (1110)	48.9 (109)	3.2	50.2 (2124)	52.1(162)	1.9
Female	47.9 (1021)	51.1(114)		49.8 (2111)	47.9 (149)	
Education of HH head						
No schooling	32.6 (695)	22.9 (51)	9.7**	34.0 (1441)	24.7 (77)	9.3**
Primary	34.5 (736)	35.4 (79)	−0.9	34.4 (1457)	41.8 (130)	−7.4**
Post-primary	32.8 (700)	41.7 (93)	−8.9**	31.6 (1337)	33.5 (104)	−1.9
Household size						
< 3	14.2 (303)	10.3 (23)	3.9	12.0 (509)	14.1 (44)	−2.1
3–5	52.2 (1113)	54.3 (121)	−2.1	45.9 (1944)	47.3 (147)	−1.4
> 5	33.6 (715)	35.4 (79)	−1.8	42.1 (1782)	38.5 (120)	3.5
Employment sector						
Unemployed	29.9 (625)	28.8 (62)	1.1	29.6 (1173)	23.1 (67)	6.5**
Agricultural	25.3 (527)	31.2 (67)	−5.9*	26.8 (1059)	32.4 (94)	−5.6**
Non-Agricultural	44.8 (935)	40.0 (86)	4.8	43.6 (1726)	44.5 (129)	−0.9
Wealth Asset Quantiles						
Lowest quartile	26.4 (563)	10.8 (24)	15.7***	30.4 (1288)	19.3 (60)	11.1***
Lower-middle	25.9 (552)	32.7 (73)	−6.8**	23.9 (1014)	20.9 (65)	3.0
Upper middle	25.6 (546)	20.2 (45)	5.4*	23.6 (1003)	33.7 (105)	−10.1**
Highest quartile	22.0 (470)	36.3 (81)	−14.3***	22.0 (930)	26.0 (81)	−4.0*
Residence						
Rural	71.1 (1516)	54.7 (122)	16.4***	70.5 (2987)	55.3 (172)	15.2***
Urban	28.9 (615)	45.3 (101)		29.5 (1248)	44.7 (139)	

The **Difference** was calculated by subtracting the proportion of the intervention group from that of the control group (Control-Intervention)

***n*** = sub-samples; 95% significance levels** *p* < 0.05, ***p < 0.01

No Difference was observed in NHIS enrollment between the two groups before intervention. However, after intervention, our findings show a 10.5% difference in NHIS enrollment between the control and intervention groups (95% CI: −15.2 to −5.7; *p* < 0.001). Comparing the control and intervention groups, before the intervention, ISWs in both groups were relatively comparable in terms of health insurance coverage, age, sex, household size, and employment sector characteristics. Our descriptive analysis, however, revealed significant differences between the two groups in regard to household head education level, wealth asset index, and location of the respondents. The intervention group was more literate, richer and resided more in urban areas compared to the control group. Post-intervention, a comparative analysis of our study sample revealed that, except for two variables (wealth asset index and household size), the intervention and control groups were comparable in terms of the other covariates before and after the intervention.

Table 4 Displays the proportions of individuals enrolled in the NHIS in the intervention and control counties before and after AC-FHC policy implementation by socio-economic characteristics. The descriptive heterogeneity analysis included 849 participants (intervention = 103 and control = 746) who were reported to be enrolled in the NHIS. In the intervention counties, the results show a significant increase in NHIS enrollment in individuals with no basic education, from 0% in 2018 to 30% in 2020 (Diff = 30.4% 95% CI: 19.3–41.6; *p* < 0.001), and among the poorest individuals, from 6% in 2018 to 20% in 2020 (Diff = 14.4% 95% CI: 1.8–27.1; *p* = 0.025). In the control group, we observed a slight increase in NHIS enrollment within the poorest quantile, from 8% in 2018 to 14% in 2020 (Diff = 6.1% 95% CI: 1.6–10.7; *p* = 0.009). We also observed that fewer people from rural areas were enrolled in 2020 than in 2018.

**Table 4:** Descriptive bivariate heterogeneity analysis among the insured ISWs by exposure group

Variables	Intervention counties			Control counties		
	**Before (2018) *N* = 34**	**After (2020) *N* = 69**	**Difference (After – Before)**	**Before (2018) *N* = 250**	**After (2020) *N* = 496**	**Difference (After – Before)**
Age						
18–34 years	44.1	34.8	−9.3	44.0	42.9	−1.1
35–60 years	47.1	47.8	0.7	49.6	49.6	−0.00
> 60 years	8.8	17.4	8.6	6.4	7.5	1.1
Sex						
Male	64.7	52.1	−12.6	68.4	65.5	−2.9
Household size						
< 3	26.5	37.7	11.2	35.6	37.3	1.7
4–6	58.8	50.7	−8.1	49.6	48.4	−1.2
> 6	14.7	11.6	−3.1	14.8	14.3	−0.5
HH head education						
No schooling	0.0	30.4	30.4***	15.2	16.4	1.1
Primary	23.6	27.6	4.0	32.0	28.0	−4.0
Post-primary	76.5	42.0	−34.5***	52.8	55.6	2.9
Employment sector						
Unemployed	24.2	17.2	−7.1	10.9	9.7	−1.2
Agricultural	18.2	26.6	8.4	23.0	21.6	−1.4
Non-Agricultural	57.6	56.3	−1.3	66.1	68.6	2.6
Wealth Asset Quantiles						
Lowest quartile	5.9	20.3	14.4**	8.0	14.1	6.1**
Lower-middle	11.8	16.0	4.2	20.8	15.0	−5.8
Upper middle	14.7	27.6	12.9	27.2	28.0	0.8
Highest quartile	67.6	36.2	−31.4***	44.0	42.7	−1.3
Residence						
Rural	47.1	53.6	6.6	65.6	56.7	−8.9**

The **Difference** was calculated by subtracting the proportion before from that after (After -Before)^a^

*HH* Household, *ISWs* Informal sector workers

95% significance levels * *p* < 0.1, ** *p* < 0.05, ***p < 0.01

^a^The 95% CI are wide due to the small sample size among the intervention group

Table 5 summarises the output from the DID logistic regression model. The results show a positive adjusted odds ratio, indicating that there was a higher propensity to enrol in the NHIS among ISWs exposed to free healthcare services. ISWs in the intervention counties had a 65% higher probability of enrolling in the NHIS post-intervention than those in control counties relative to the pre-intervention period (odds ratio −1.65; *p* = 0.07; 95% CI: 0.96- 2.83). The difference was, however, marginally statistically significant (*p*-value = 0.07; 95% CI: 0.96–2.83). A detailed Table showing the model results and covariates is available in the Annexe section as Table 6.

**Table 5:** Difference-in-differences logistic regression estimates for health insurance enrollment

	**Odds Ratio**	**(95% CI)**	**Standard Error**
Free Health Care Policy			
Control	1		
Intervention	2.29	(1.02–5.17)	0.95**
Time			
Before (2018)	1		
After (2020)	1.11	(0.86–1.43)	0.14
Time * Free Health Care Policy			
Interaction term	1.65	(0.96–2.83)	0.45*
Number of observations: 6550			

*CI* Confidence Interval, 95% Significance Levels * *P* < 0.1, ** *P* < 0.05, ***P < 0.01; the 95% CI are wide due to the samll sample size among the intervention group

## Discussion

Our study makes an important contribution to the literature as the first study to examine the effect of a free healthcare policy implemented outside NHIS structures on an individual's decision to enrol in a national health insurance program. The value of our research lies in its emphasis on ISWs whose inclusion in the NHIS is critical for achieving UHC in Kenya and elsewhere in low- and middle-income countries. While we recognize the limitations of contributory health insurance schemes, such as low coverage, adverse selection, and inadequate financial protection for the poor [12, 19, 67], we trust in the value of our research, as it is important, in this intermediate policy period, to understand how free healthcare policies shape informal sector workers’ decisions to join existing health insurance programs.

Our findings indicate that the propensity to enrol in the NHIS was higher in counties that had exposure to the AC-FHC pilot compared with counties that had not, with individuals in intervention counties having 1.65 times the odds of being enrolled in the NHIS compared with individuals in control counties. Our findings are consistent with the free trial conceptual model, whereby individuals who experience a service for free are more willing to later pay for that service or its replacement, as they appreciate its value during the free trial period [49, 50]. We postulate that the AC-FHC policy provided the opportunity to access healthcare services at no cost at the point of care to a population who otherwise would forgo care due to financial barriers, limited availability of quality services, and mistrust in healthcare [68, 69].

The findings suggest that, after removing the AC-FHC policy, ISWs who had experienced the benefit of accessing care at no cost at the point of use and could pay premiums enrolled in the NHIS, the most affordable available option for maintaining these benefits while protecting themselves from costly healthcare fees. From a policy perspective, our findings show that brief exposure to free or subsidised policies may incentivise NHIS enrollment among ISWs who can afford to pay premiums. For countries considering a transition to the NHIS and those seeking to enforce mandatory enrollment, it may be valuable to reflect on the potential benefit of short-term exposure to free or subsidized services as a preparatory step to allow ISWs with the capacity to pay premiums to experience, value and build trust with the system and be motivated to enroll in NHIS schemes. The policies can vary depending on the population characteristics.

Since no other study has assessed the effect of stand-alone free healthcare policies such as AC-FHC on ISWs' decision to enrol in NHIS, various studies that have evaluated subsidies within health insurance structures present the closest opportunity for comparison. An experimental trial study in Indonesia found that full but temporary one-year health insurance premium subsidies, coupled with registration assistance and information sessions, increased enrollment by 30% [34]. Studies in Kenya and Ghana showed that small subsidies of 2%, 10%, or 30% do not motivate enrollment [23, 45]. The Kenyan study further revealed that a full subsidy only generated 45% enrollment, with no retention after a year [23]. In Vietnam, information campaigns and subsidies had limited effects on voluntary health insurance enrollment [46].

Two studies further reported that subsidies alone were insufficient to achieve universal coverage among ISWs, an observation we also make in our findings. They further suggest that combining interventions yielded better outcomes [34, 45], a consideration policymakers should consider when designing strategies to address the diverse needs of ISWs. While premium subsidies alone may not be enough to ensure that all individuals join insurance [34, 45], evidence from other settings has shown their potential to increase coverage among the poor population, especially if incorporated in the existing NHIS structures. A study in Ghana reported a high retention rate within the scheme when a premium subsidy was provided to low-income individuals already enrolled, preventing them from dropping off [44]. Currently, in Kenya, NHIS premium subsidies are limited to poor and vulnerable elderly individuals, disabled children, and all pregnant women [14, 16, 26, 28]. We recommend that policymakers consider expanding premium subsidisation, partially or in full, within the limits of their financial capacity to support low-income informal sector workers currently not targeted through any of the specific programs.

Our findings demonstrate that the intervention group experienced a significantly higher enrollment rate (10.5%) compared to the control group. While this increase is notable, it's lower than the 30% rise reported in the Indonesian trial [34] and the 45% observed in a Kenyan study [23]. Both studies involved full subsidies for NHIS premiums for one year. On the contrary, the AC-FHC policy was implemented outside the NHIS structures, which may partly explain the differences in enrollment rates. This evidence suggests that integration within existing insurance systems, where they already exist, can significantly influence enrollment outcomes.

Despite the improvement in intervention counties, we note that NHIS enrollment has, in practice, remained low among ISWs; our findings show that only two out of ten people are insured. This rate is worrisome, considering that informal sector workers represent 84% of the Kenyan employed population and the main target group to ensure that the country achieves UHC [29]. In Kenya, the low enrollment rate can be attributed to the unaffordability of the flat rate premium (KES 500; USD 3.85), with the majority of the ISWs willing to pay just half of the current rate [7, 12, 14]. Other contributing factors include the complexities of inadequate information [16, 70] and the voluntary nature of enrollment practice among ISWs despite the enactment of a mandatory NHIS enrollment policy [27, 33]. Further evidence from other settings indicates that premium costs and expenses related to the enrollment process contribute to reducing enrollment rates or increasing dropout rates among ISWs [34, 48, 71, 72]. If mandatory enrollment for ISWs is not enforced in practice by subsidising premiums and by instituting facilitative structures [34, 36, 48], enrollment in the NHIS will remain low, as only ISWs with the capability to pay may be onboard [67].

Recognising the inability to expand subsidies to cover the premium fully for all informal sector workers, we also acknowledge the need to increase awareness and improve individual motivation to enrol in the NHIS among those who can afford to pay. Here, we point to the potential to engage in information and communication campaigns that use channels other than traditional media, such as the widely established Community Health Volunteers/Promoters network [73]. A study in Nepal highlighted the female CHVs' key role in health insurance scheme awareness [74]. Additionally, partnerships could be built with existing microfinance institutions [48], exploiting their high penetration rates in the informal sector in Kenya by leveraging the structures and trust within the institutions while tapping into ISWs with the capacity to pay premiums, thus facilitating NHIS enrollment [75]. These partnerships could enhance awareness and have the potential to attract more healthy ISWs into the scheme, balancing the pool and making it more sustainable [23, 48].

Finally, we note how our further descriptive heterogeneity analysis showed that exposure to the AC-FHC policy had the potential to improve equity among the insured, with enrollment rates increasing proportionally more among the poorest and uneducated individuals in the intervention compared with control districts. Although this was not the focus of our paper, the registrations of people in their neighbourhoods during the registration of the Afya Care-free Health Policy reached those who remained unreached. Their experience during the registration motivated their enrollment.

## Methodological limitations

Despite the innovative nature of our analysis, we acknowledge a few methodological and data limitations. Our study relied on repeated cross-sectional data to estimate the effect of the AC-FHC policy on individuals’ decisions to enrol in the NHIS. As such, our analytical model relied on a quasi-experimental approach. A lack of randomisation could affect our study's internal and external validity due to the non-random allocation of the intervention. However, in the absence of controlled experimental data, the strength of our approach lies in using nationally representative data to advance knowledge on the association between FHC policies and enrollment in national insurance schemes. Additionally, this study relied on a secondary dataset, which did not include all possible variables we might have wished to include as covariates, such as health status, number of visits, and distance to health facilities. We recognise that adjusting for additional covariates might have improved the precision of our estimates to an extent that our current model could not do. Yet, we acknowledge that it would not change the direction of the estimates and hence would not really shape our narrative differently. One could also criticise the small sample sizes in the intervention group compared with the control group, which increases the risk of bias and confounding variables influencing our results. We took caution through various robustness checks and sensitivity analyses to ensure the reliability of our findings. First, common trend analysis showed that health insurance enrollment in the two groups was similar over time before the intervention. Second, we controlled for individual, household, and county cluster-specific effects.

## Conclusion

In conclusion, the empirical evidence provided in our paper suggests that the experience of the free healthcare policy in Kenya influenced ISWs' decisions to enrol later in the NHIS. Our findings further suggest that exposure to free healthcare policy has the potential to improve equity among the insured. Still, we recognise that enrolment rates remain low because, despite being mandatory in principle, enrolment remains voluntary in practice. Experience alone is not sufficient for everyone to become covered. To increase enrolment rates while working to ensure that enrolment becomes mandatory in the long run, policymakers should consider introducing subsidies to increase premium affordability, especially among low-income ISWs, and to subsidise fully those who cannot pay premiums. Recognising the diverse heterogeneity, subsidies should be combined with other strategies, such as improving communication campaigns targeting less educated populations to boost awareness. Our study findings are particularly timely for health financing policymakers in Kenya, who are currently working on the development of policies aimed at including informal sector workers in the NHIS. The results also apply to other Low and middle-income countries within and beyond the sub-Saharan region that are considering the expansion of the NHIS to informal sector workers.

## Supplementary Information

Supplementary Material 1.

## Data Availability

The datasets generated and/or analysed during the current study are available in the repository *Dataset on Informal Employment, Social Security and Political Trust in six sub-Saharan African Countries/Friedrich-Ebert-Stiftung, German Institute of Development and Sustainability, International Labour Organisation.—(Version 1.0): Friedrich-Ebert-Stiftung. – 2023.

## Annexes

**Table 6:** Detailed DID regression output

Health insurance coverage	Model1: Unadjusted	Model 2: Adjusted
	***OR*** ** (95% C.I)**	***OR *** **(95% C.I)**
Free health policy (ref. control)		
Intervention	1.35 (0.79-2.31)	2.29** (1.02-5.17)
Time (ref. before-2018)		
After (2020)	0.99 (0.66-1.49)	1.11 (0.86-1.43)
Interaction term		
Time*AC-FHC	1.59 (0.72-3.51)	1.65* (0.96-2.83)
Age (ref. 18-34 years)		
35-60 years		1.76*** (1.48-2.09)
>60 years		2.25*** (1.59-3.18)
Sex (ref. Male)		
Female		0.58*** (0.49-0.69)
Household size (ref. <2)		
3-5		0.83 (0.64-1.07)
>5		0.61** (0.44-0.87)
Wealth asset index (Ref. Lowest quartile)		
Lower-middle		1.26 (0.94- 1.69)
Upper middle		1.92*** (1.45-2.54)
Highest quartile		3.34*** (2.41-4.64)
HH head education (ref. No schooling)		
Primary		1.10 (0.85-1.42)
Post-primary		2.01*** (1.49-2.70)
Employment (ref. Unemployed)		
Agricultural		1.71** (1.22-2.38)
Non-Agricultural		3.45*** (2.67-4.45)
Residence (ref Urban)		
Rural		0.79 (0.59-1.06)

Number of observations: 6550; HH=household head; OR= Odds Ratio; CI= Confidence interval; 95% significance levels * p<0.1, ** p<0.05, ***p<0.01; Model 2 includes county dummies

## Abbreviations

AC-FHC: Afya Care Free Health Care
CHV: Community Health Volunteers
DID: Difference in Difference
FHC: Free Health Care
IDOS: Institute of Development and Sustainability
ISWs: Informal Sector Workers
KES: Kenyan shillings
KEMSA: Kenya Medical Supplies Agency
NHIS: National Health Insurance Scheme
SSA: Sub-Saharan Africa
UHC: Universal Health Coverage
USD: US Dollars

## Footnotes

1 . Since the introduction of a health insurance scheme in 1960, the National Health Insurance Scheme (NHIS) had been publicly known as the National Hospital Insurance Fund (NHIF). The official name changes to NHIS happened in 2022 following amendments to the NHIF Act.

2 Makueni County was excluded from the analysis as it had an existing extensive county health insurance scheme before the launch of the AC-FHC pilot, whose inclusion would bias the results.
