# Supplementary material for: Do free healthcare policies play a role in expanding national health insurance enrollment among informal sector workers? The case of the Afya Care pilot program in Kenya
Source: BMC Public Health. 2025 Oct 27;25:3610. doi: 10.1186/s12889-025-24760-3 (PMC12560322; doi:10.1186/s12889-025-24760-3)
Supplement: Supplementary file 1 — Supplementary Material 1. [file 12889_2025_24760_MOESM1_ESM.docx]

**Sampling Design and Process**

The sample universe associated with our survey includes all households in Kenya that operate in the informal economy on the day of the survey. We exclude households that are operating in the formal economy. To obtain a nationally representative cross section of this target population, we use the most recent national census data from the National Bureau of Statistics (KNBS) in Kenya. We used a clustered, stratified, multi-stage, probability sample design. The objective of our sample design was to give every household that operates in the informal economy an equal chance of being chosen for inclusion in the sample. This ensures that the survey provides a representative estimate of the views of the target population. We reached this objective by (a) strictly applying random selection methods at every stage of sampling and by (b) applying sampling with probability proportionate to adult population size.

The sampling process was based on stratification of the country into regions. Regions were further classified into counties, and these were further divided into districts and villages. Primary sampling units (PSUs)—sometimes referred to as enumeration areas—are the smallest geographical unit/cluster for which reliable population data were obtainable. The primary sampling units were selected from each stratum based on shares of the national population and number of households, and further allocated based on the urban/rural divide.

The sampling process was structured in four stages and follows largely the process of the Afrobarometer surveys (Afrobarometer Survey Manual [2017](https://link.springer.com/article/10.1057/s41287-022-00541-1#ref-CR4)): (i) selection of enumeration areas; (ii) selection of sampling start points; (iii) selection of households; and (iv) identifying households that operate in the informal economy for interview.

(i) Selecting enumeration areas (EA): Based on the latest and updated population census Kenyan National Bureau of Statistics (KNBS) randomly select enumeration areas for each stratum and respective rural/urban divide, based on probability proportional to size of population and number of households.

(ii) Selecting the sampling start points (SSPs) for each enumeration area: As no complete lists of households of the informal economy were available from which the sample could be randomly drawn for each EA, we use physical maps of the enumeration areas that were provided by the KNBS. A random sampling start point (SSP) is marked on the map and field teams travel as close as possible to it, or to housing settlements nearest to it. A second SSP is selected as a reserve or substitute in case the initial SSP is inappropriate or inaccessible. Random selection of a start point uses a grid. A ruler is placed along the top of the map and another along the side. A table of random numbers is then used to select pairs of numbers, one for the top axis and one for the side axis, resulting in a random combination. A line is then drawn on the map horizontal to the number chosen on the side, and another line is drawn vertical to the number chosen on the top. The point on the map where these two lines intersect is the sampling start point. Each *x*–*Y* pair of numbers from the random number table can be used only once.

(iii) Selecting the household—walking pattern of interview teams: The interviewers start walking away from the physical startpoint, with interviewer 1 walking towards the sun; interviewer 2 in the opposite direction; interviewers 3 and 4 at a 90-degree angle to the right and left. With this walking pattern, all four directions are covered. By counting households on both sides of the walking path, household No. 5 is selected as the first household for the interview and household No. 15 for the second interview. Special rules were applied in the case of multi-storey buildings, widely scattered households and settlements within commercial farms.

If the interview cannot take place because nobody is at home, or the interview starts but cannot be finished, the walk continues to the next household on the same side of the road or opposite (household No. 6), while the second interview is done in household No. 16.

If the interview is refused the walk continues in the same direction until household No. 15. The second interview would take place with household No. 25.

(iv) Identifying households for the interview that operate in the informal economy: At the household level, each interview is done in two phases. Phase 1 of the interview is conducted with the household head living in the household. The household head provides demographic and employment information on each member of the household (15 or older).

Based on this screening a list is drawn up to include all household members who operate in the formal and informal economy. The interview was ended if at least one member (15 or older) is active in the formal economy and the household was replaced by another household.

For households were no member is active in the formal economy, the respondent for the main part of the interview (phase 2) is randomly selected from the list of persons that operate in the informal economy for interview. If the randomly selected respondent is unavailable the fieldworker makes an appointment for a later time in the day for a second attempt. If the interview is unsuccessful after the second attempt, the fieldworker randomly selects another respondent who qualifies within the same household for the interview. If the second respondent is unavailable or the interview is unsuccessful for whatever reason, the household is dropped and the fieldworker replaces it with another household.

To identify activities within the informal economy, the survey used the following operational definitions: (i) Informal farming, raising animals or fishing: economic activities whose products have been produced for sale were grouped as informal. (ii) Informal employees: paid job with no reference to an employer’s tax contribution or contribution to a public or private pension scheme. If employers did not pay contributions, employees were grouped as informal. (iii) Informal employers and own-account workers: informality is defined by non-registration in the national registry, which is used for company taxation. (iv) Contributing family workers: defined, by default, as having an informal job because of the informal nature of jobs held by contributing family workers that also can include unemployed or students.
